# Blind Fault Extraction of Rolling-Bearing Compound Fault Based on Improved Morphological Filtering and Sparse Component Analysis

**DOI:** 10.3390/s22187093

**Published:** 2022-09-19

**Authors:** Wensong Xie, Jun Zhou, Tao Liu

**Affiliations:** Faculty of Mechanical and Electrical Engineering, Kunming University of Science and Technology, Kunming 650500, China

**Keywords:** morphological filtering, density peak clustering, orthogonal matching pursuit, sin*C* function, sparse component analysis

## Abstract

In order to effectively separate and extract bearing composite faults, in view of the non-linearity, strong interference and unknown number of fault source signals of the measured fault signals, a composite fault-diagnosis blind extraction method based on improved morphological filtering of sinC function (SMF), density peak clustering (DPC) and orthogonal matching pursuit (OMP) is proposed. In this method, the sinC function is used as the structural element of the morphological filter for the first time to improve the traditional morphological filter. After the observation signal is processed by the improved morphological filter, the impact characteristics of the signal are improved, and the signal meets the sparsity. Then, on the premise that the number of clustering is unknown, the density peak algorithm is used to cluster sparse signals to obtain the clustering center, which is equivalent to the hybrid matrix. Finally, the hybrid matrix is transformed into a sensing matrix, and the signal is transformed into the frequency domain to complete the compressive sensing and reconstruction of the signal in the frequency domain. Both simulation and measured signal results show that this algorithm can effectively complete the blind separation of rolling bearing faults when the number of fault sources is unknown, and the time cost can be reduced by about 75%.

## 1. Introduction

As rolling bearings are usually installed in the key position of rotating machinery, mechanical failures are often caused by lubrication, manufacturing errors and unreasonable forces [1,2]. In actual operation, bearing faults such as cracks, pits and spalling [3] often occur simultaneously or in succession, which will produce group faults or multi-point composite faults. This means that in the actual industrial manufacturing site, what is collected by the sensor is often not a single vibration source, but the coupling of multiple signals [4]. The above phenomena make troubleshooting very difficult. Therefore, in order to solve this problem and improve the accuracy of modern equipment fault detection and diagnosis, the most critical step is to separate the fault signal from the mixed signal.

Scholars generally prefer to study composite faults from the perspective of the analysis model and signal processing, respectively. The former studied the vibration response of the fault excitation from the mechanism and established the dynamic model to simulate the composite fault. For example, Wu et al. [5] completed the robust diagnosis of stator/rotor winding early faults by establishing the dynamic model of a squirrel caged induction motor. Patel et al. [6] studied the vibration response characteristics of bearing inner and outer ring surfaces with single and multiple faults. Wang et al. [7]. studied the dynamic characteristics of deep groove ball-bearing composite faults through model analysis, and the results show that the vibration response of composite faults is the result of the coupling effect of the vibration response of inner and outer ring faults.

The signal processing method is mainly to analyze the information collected by the sensor. In the actual working condition, the signal picked up by the sensor is the result of the coupling of multiple faults. Blind source separation (BSS) [8] technology can separate multiple signal sources from mixed signals when the transmission channel is unknown. In recent years, the continuous development of fault diagnosis technology based on blind signal processing provided new ideas and means for solving this problem. Generally speaking, there are two methods to solve BSS by a signal processing method, which are independent component analysis (ICA) [9,10] and sparse component analysis (SCA) [11].The former decomposes the collected observation signals into multi-channel modal signals and constructs the input matrix of ICA through these modal signals. Related algorithms are ensemble empirical mode decomposition (EEMD) [12,13,14] or variational mode decomposition (VMD) [15,16,17]. The latter mainly probed into the mixing matrix between the signal source and the observed signal. With the development of modern sparse methods, if the sparsity of observation signals is sufficient, the BSS problem can be regarded as the estimation problem of the mixed matrix [18].

The premise of the ICA algorithm is that the source signals are statistically independent, and each independent component must conform to a non-Gaussian distribution [19]. However, it is difficult for modern machinery and equipment to meet the hypothesis of statistical independence [20,21,22]. In contrast, the sparsity assumption of SCA is relatively effortless to satisfy. In a comprehensive comparison, SCA is more suitable as a method to solve BSS. 

In the research of SCA algorithm, the selection of the mixing matrix, that is, the estimation of the number of sources, is an intractable problem. The clustering method is the preferred solution to this problem. For example, Wang et al. [23] proposed an effective two-stage clustering algorithm, thereby improving the estimation accuracy of the hybrid matrix. He et al. [24] proposed an algorithm based on the improved K-means clustering algorithm and Laplace potential function to estimate the mixing matrix. However, the potential function method is sensitive to the division interval, and the anti-interference ability to noise is not strong enough. In 2014, Rodriguez et al. [25] proposed a density peak clustering algorithm. This algorithm can intuitively obtain the number of sources, and it is easy to find outliers with single parameters and good robustness. There is a certain potential in dealing with the estimation of the number of vibration sources. For example, Lu et al. [26] combined synchronous compression transformation with density peak clustering to achieve blind source separation with an unknown number of sources. Li et al. [27] proposed a new hybrid matrix estimation method based on single-source point detection and density peak clustering. Hu et al. [28] used density peak clustering to achieve effective mode estimation without knowing the number of effective modes.

The research of the SCA algorithm also includes the recovery of source signal. The mainstream methods of source signal recovery can be divided into two categories. One is to recover the source signal by optimizing the function approximating the L0 norm. For example, the smooth continuous function is used to approximate the L0 norm, which is called the smooth L0 norm method [29,30]. Zhang et al. [31] approximated the L0 norm with compound trigonometric functions. However, when the incoming direction of the source signal is closer, the recovery accuracy will decrease. Another method is to use the compressed sensing method [32], which uses L1 norm optimization instead of L0 norm optimization to restore the source signal, avoiding the L0 norm optimization NP-hard problem. In 2007, Tropop et al. [33] proposed the orthogonal matching pursuit algorithm (OMP), which plays an important role in the research of reconstruction algorithms. Pala et al. [34] adopted OMP to reduce the performance complexity of devices such as analog-to-digital converters. Zhang et al. [35] proposed an OMP algorithm based on improved singular value decomposition, which effectively reduced the correlation between the measured values.

Combined with the above research content, this paper presents a blind source separation method for bearing compound faults, which combines modified morphological filtering based on sinC structural elements (SMF), density peak clustering (DPC) [25] and orthogonal matching pursuit (OMP) algorithm, called SMF-DPC-OMP. It is mainly employed to realize blind separation of compound faults when the number of fault sources is unknown, which has certain reference significance for fault extraction in practical production. Firstly, the modified morphological filter is used to de-noise the observed signal. While promoting the signal-to-noise ratio, the impact component of the signal is highlighted. Secondly, the filtered signal is processed by density peak clustering to obtain the clustering center, which also is the sensor matrix. Finally, the filtered signals are converted to the frequency domain to meet the sparsity requirements, and the source signals are reconstructed using OMP algorithm to estimate the source signals of bearing compound faults. In addition to improving computing speed and adaptability, it realizes fault feature extraction. The validity and accuracy of the proposed algorithm are verified by simulation and actual vibration signal extraction of rolling bearing complex faults. The main contributions of this work are as follows:

(1) A blind extraction method for complex fault diagnosis based on sin*C* function improved morphological filtering (SMF), density peak clustering (DPC) and orthogonal matching pursuit (OMP) was proposed;
(2) In terms of morphological filtering, a new structural element based on sin*C* function is proposed, and its performance is higher than that of the traditional linear structural element;
(3) The DPC algorithm is used to overcome the problem that the number of fault signal sources is difficult to determine in actual fault diagnosis. The parameters of the algorithm will not change with the change of signal ratio, so it has a certain robustness;
(4) By replacing linear programming with OMP algorithm, the time cost of the algorithm is greatly reduced while the sparsity of the signal is improved;
(5) Compared with the traditional blind source separation algorithm and modern signal decomposition method, the proposed method can efficiently complete blind source separation of complex faults, and the spectral clarity is also improved to some extent.

The overall narrative structure of this paper is as follows. The theories and concepts of BSS and morphological filtering (MF) are introduced in Section 2 and Section 3, respectively. Section 4 explains SMF−DPC−OMP proposed in this paper. Section 4.1 mainly describes the parameter selection of structural elements of the improved morphological filter, and the idea and process of density peak clustering algorithm is stated in Section 4.2. Section 4.3 demonstrates how to carry out frequency domain compressed sensing and reconstruction by OMP, and Section 4.4 reveals the overall process of the SMF−DPC−OMP algorithm. The simulation experiment in Section 5 verifies the feasibility of the algorithm. In Section 6, the actual vibration signals of rolling bearings are analyzed. Section 6.1 compares the algorithm in this paper with other algorithms to analyze its advantages. Section 6.2 tests the performance of the proposed algorithm under different SNR. There is a brief summary of the advantages and disadvantages of this method in Section 7. Section 8 is the conclusion of the whole paper. 

## 2. Mathematical Model of Blind Source Separation

The blind source separation of the technique of recovering and estimating the source signal using only the observed signal when the signal transmission channel and source signal are unknown [36,37]. The mathematical model of SCA method containing noise is:

(1) $$X_{m\times t}=A_{m\times n}S_{n\times t}+V_{m\times t}$$

where X is the observation matrix, namely the actual signal value collected by the sensor; A is the mixed matrix, which needs to be solved by algorithm; S is sparsely distributed unknown source signal; V is noise or other random interference components; m is the number of observation signals picked up by the sensor, n is the number of unknown source signals, and m<n is underdetermined; t is the observation time.

## 3. Morphological Filtering

MF is a nonlinear filtering method based on ensemble theory, which can approximate some noise to zero while retaining the main characteristics of the signal. To a certain extent, it satisfies the requirement of sparsity in the mixing matrix phase of sparse component analysis estimation. The current way of applying mathematical morphology to fault feature extraction is mainly to construct morphological filters, which are used to extract and highlight the impact components in the signal. The feature extraction of the signal is carried out in the time domain by this method. Compared with the traditional filtering method, the algorithm is simpler, the calculation speed is faster, and has the advantages of easy hardware implementation.

The quality of MF mainly depends on the selected structural elements and morphological transformation operations. The selection of structural elements includes elements such as the shape, length, and height of structural elements (the amplitude of structural elements). The shapes of structural elements generally include linear, triangular, semicircular, sine and so on. The basic operations of morphological transformation in contain corrosion, expansion, morphological open operation and morphological closed operation.

In one-dimensional signal processing, mathematical morphology mainly includes morphological corrosion, morphological expansion, morphological open and morphological closed operators. Θ, ⊕, ∘ and • are the operators of corrosion, expansion, open and closed operations, respectively. The definition is as follows: 

Suppose the original signal f(n) and the structural element g(m) are discrete functions on F(1,2,…N−1) and G=(1,2,…M−1) respectively, N≥M, then the expansion and corrosion of f(n) with respect to g(m) are defined as [38]:

(2) (f⊕g)(n)=max[f(n−m)+g(m)]

(3) (fΘg)(n)=min[f(n+m)−g(m)]

Open and close operations are defined respectively:

(4) (f∘g) (n)=(fΘg⊕g)(n)

(5) (f•g) (n)=(f⊕gΘg)(n)

The cascade form of morphological open and morphological closed is usually used to remove the positive and negative noises in the signal. The structural elements of the same size are uses to define close-open (*CO*) and open-close (*OC*) filters:

(6) CO (f(n))=(f•g∘g)(n)

(7) OC (f(n))=(f∘g•g)(n)

In order to suppress statistical bias, the morphological *CO* operator and *OC* operator are usually combined to form the average combined filter [38]:

(8) y(n)=[OC(f(n)+CO(f(n)]/2

## 4. Blind Extraction of Compound Faults Based on SMF−DPC−OMP

**Figure 1:**
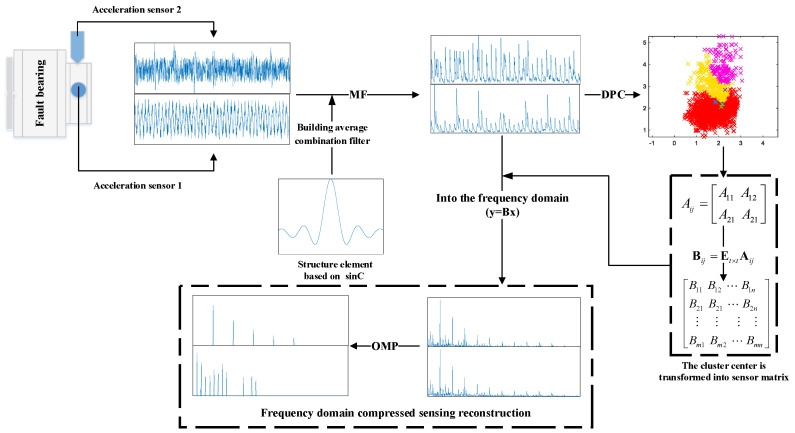
Flow chart based on SMF−DPC−OMP.

Combining the advantages of the above analysis and research, this paper adopts an improved morphological filter, density peak clustering and frequency-domain compressive sensing reconstruction algorithm to blindly extract bearing composite fault sources. The basic process is shown in Figure 1. For the three pivotal links of the process, Section 4.1 describes how to create a morphological filter based on sinC function; Section 4.2 introduces how to use the clustering method to estimate the mixture matrix; Section 4.3 demonstrates how to reconstruct and recover source signals through compressed sensing, and blind separation equivalence relation is introduced in detail, and Section 4.4 is a description of the overall algorithm of this paper.

### 4.1. MF Based on sinC Structural Elements

The sinC function, also known as the Singer function, is represented by sinC(x). This function is defined as follows:

(9) $$\sin C\left(x\right)=\frac{\sin\left(\pi x\right)}{\pi x}$$

**Figure 2:**
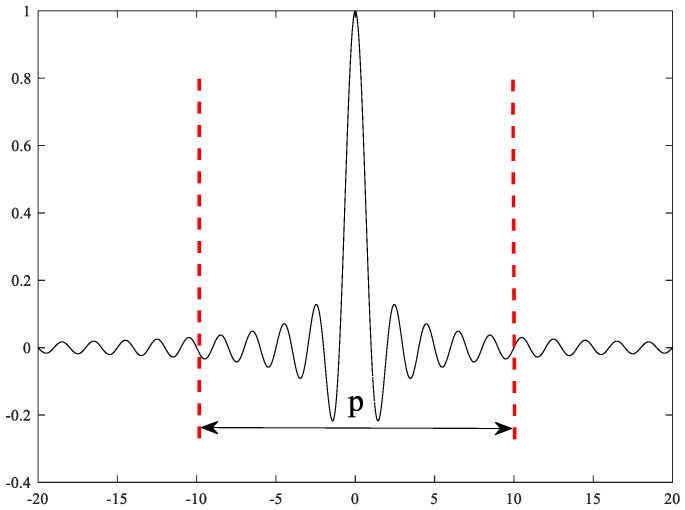
The parameter diagram P (main lobe ratio) of sinC.

In this chapter, the sinC function is used as the structural element of MF to construct an average combined filter, and a new MF method is proposed. In addition, two parameters are mainly defined when sinC is used as a structural element, namely, the length L and the main lobe ratio P. Length refers to the length of the entire sinC image, and the main lobe ratio refers to the percentage of the entire image taken from the middle to the sides. For instance, Figure 2 shows a sinC graph with L at 20 and P at 50%.

**Figure 3:**
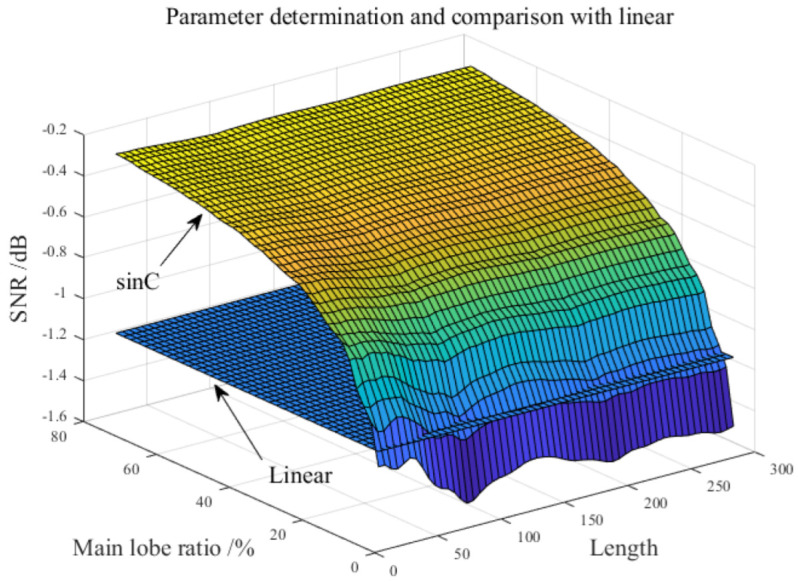
The parameter selection and its influence on filtering effect and compared with linear.

Two main things have been accomplished in Figure 3:

(1) In order to obtain the influence of sinC structural element parameters on filtering effect, the failure test bench was used to collect the data of the inner and outer ring failures. The results of the new filter are shown in Figure 3.

(2) So as to verify the advantages of the proposed new structural element, the filtering effect of the traditional linear structural element under optimal parameter selection is compared (Li et al. verified that when the length of linear structural element is 10, the performance is the highest [39]).

It is not difficult to find from Figure 3 that the overall filtering performance of sinC is better than linear filtering. The main parameter affecting the filtering effect is the main-lobe ratio, while the length has little influence, which can be easily found in Figure 3. It is worth noting that the ratio range of the main lobe is 5% to 80%, because when parameters are selected outside this range, serious distortion will occur to the filtered signal. It should not be forgotten that even if the main lobe ratio is selected within the range, distortion cannot be completely avoided. To sum up, sinC functions with length of 90 and main lobe ratio of 20% are selected as structural elements after several experiments, and the average combined filter is constructed according to this structural element and Section 2.

### 4.2. The Theory of Density Peaks Clustering

The DPC algorithm mainly follows two assumptions: (1) The density of the cluster center itself is greater than that of its neighbors, that is, the cluster center is surrounded by low-density points; (2) The distance between the cluster center and the sample point with higher density is relatively large.

There is a data set $X=\left\{x_{1},x_{2},\cdots x_{N}\right\}$, $x_{i}={\left(x_{i1},x_{i2},\cdots x_{ip}\right)}^{T},i=1,2,\cdots N$, and $x_{ij}$ represents the j dimension attribute value of the i data point. 

The DPC algorithm consists of three steps: (1) calculating the local density of the sample point, (2) calculating the distance between the point and the nearest neighbor with higher density as the cluster center, and (3) clustering.

Since this algorithm only needs clustering centers, the first two steps are required merely.

#### 4.2.1. Calculate Local Density ρ

For each data point $x_{i},i=1,2,\cdots ,N$, the local density $\rho_{i}$ can be calculated as follows:

(10) $$\rho_{i}=\sum_{j,j\neq i}\chi \left(d_{ij}-d_{c}\right)$$

where *χ* is an indicative function, $d_{ij}<d_{c}$ is 1, and vice versa 0. $d_{ij}=dist\left(x_{i},x_{j}\right)$ represents distance between two data points, usually the Euclidean distance. $d_{c}$ is called truncation distance. Local density *ρ*_*i*_ can be thought of as equivalent to the number of points $x_{i}$ that are less distant $d_{c}$ from a point. Truncation distance $d_{c}$ is the only variable in the algorithm. In the original algorithm, the determination method is as follows:

(11) $$d_{c}=d_{N_{d}*p}$$

where $N_{d}=C_{N}^{2}$ represents the number of sample pairs, $d_{N_{d}*p}\in D\left[d_{1},d_{2},\cdots d_{{}_{N_{d}}}\right]$. *D* is the set of distances between two samples, in ascending order, N represents the sample size, $d_{N_{d}*p}$ represents the distance $d_{N_{d}*p}$ in the set D, p is 1–2% of the total sample points.

#### 4.2.2. Cluster Center Selection Based on Nearest Neighbor Distance δ

The nearest neighbor distance of a point is defined as:

(12) $$\delta_{i}=\left\{ \begin{array}{l} \underset{j:p_{j}>p_{i}}{\min\left(d_{ij}\right)},\;\rho_{i}<\max\left(\rho \right)\\ \underset{j}{\max\left(d_{ij}\right)},\;\rho_{i}=\max\left(\rho \right) \end{array}\right.$$

That is, for the non-maximum density sample points, the distance between the point i and the nearest neighbor of higher density is calculated. For the highest density point, calculate the distance between the point i and the farthest point.

When the clustering object is bearing fault signal, most of the calculated results of Equation (12) are 0. To ensure that the correct clustering centers are not omitted, the data points calculated in Equation (12) that are more than twice the mean value are determined as clustering centers. 

### 4.3. Frequency Domain Compressed Sensing (CS) Reconstruction Algorithm

The sparsity of the signal, the design of the sensor matrix, and the signal reconstruction are the basic components of compressed sensing theory. Signal sparsity reflects the degree of energy concentration of the signal itself or under a certain basis and is often measured by sparsity. The speed and precision of signal reconstruction are closely related to the sparsity of the signal, and the sparse representation of the signal is the premise of the application of compressed sensing. In this paper, FFT transform is used to transform the signal into frequency domain to meet the sparsity requirement.

By establishing the equivalence relation between compressed sensing and blind source separation, the OMP algorithm of the compressed sensing reconstruction algorithm is used to reconstruct the source signal. For the compressed sensing model, one-dimensional mixed signals are firstly constructed. Then m observation signals with length t can be transformed into $y={\left(y_{11},y_{12},\cdots y_{1t},\cdots ,y_{m1},y_{m2},\cdots y_{mt}\right)}^{T}$.

The estimation matrix of density peak clustering is used to construct the sensor matrix W. According to the compressed sensing model, when mixed signals y=(mt×1), its sensing matrix is W=(mt×nt). Using Fourier transform orthogonal matrix $E_{t\times t}$ to expand the elements of the estimation matrix A. The transformation relation is $B_{ij}=E_{t\times t}A_{ij}$. The specific transformation is shown in Equation (13):

(13) $$y=\left[\begin{array}{llll} B_{11} & B_{12} & \cdots & B_{1n}\\ B_{21} & B_{22} & \cdots & B_{2n}\\ \vdots & \vdots & \vdots & \vdots \\ B_{m1} & B_{m2} & \cdots & B_{mn} \end{array}\right]x$$

The dimension of $x={\left(x_{11},x_{12},\cdots ,x_{1t},\cdots ,x_{n1},x_{n2},\cdots ,x_{nt}\right)}^{T}$ is (*nt* × 1).

Until now, the reconstruction model of blind source separation has been completely established.

The OMP algorithm flow is a commonly used reconstruction algorithm for compressed sensing. The OMP algorithm first performs Schmidt orthogonalization on all selected atoms in each iteration process to ensure that the result of each cycle is the best solution. Constructing the frequency domain sensing matrix is the core idea of using the OMP algorithm for reconstruction here. The core algorithm steps are as follows:

(1) Initialization parameters, initial residuals $r_{0}$, number of iterations, Fourier orthogonal transformation matrix $E_{t\times t}$ is calculated, the sensor matrix W=(mt×nt) is constructed according to the formula $B_{ij}=E_{t\times t}A_{ij}$, and the signal is transferred to the frequency domain for operation;

(2) The inner product method is used to calculate the projection coefficient of the column vector and residual of the sensor matrix, and the corresponding position **β**_*i*_ of the maximum projection coefficient is recorded;

(3) The least square method is used to calculate the estimated value *x_i_* = (**β**_*i*_^*T*^•**β**_*i*_)^−1^•**β**_*i*_^*T*^•**r**_*i*_ of reconstructed signal for this iteration;

(4) Update the residuals $r_{i+1}=r_{i}-x_{i}$ and repeat step (2) until the end of the iteration.

(5) Using $E_{t\times t}$ inverse Fourier transform reconstruction to obtain the dimension (nt×1) of time domain signal x.

### 4.4. General Flow of SMF-DPC-OMP Algorithm

(1) Signal preprocessing: morphological filtering processes the observation signal, extracts the impact signal of bearing characteristics and suppresses noise. Before filtering, the structural element is constructed as described in Section 4.1 and then the filter is constructed as described in Section 3;

(2) Estimate the mixture matrix: the DPC algorithm described in Section 4.2 is used to solve the hybrid matrix;

(3) Source signal reconstruction: the hybrid matrix of step (2) is employed to construct the sensor matrix, and the source signal was reconstructed in the frequency domain according to the OMP algorithm described in Section 4.3;

(4) Fault identification: Perform FFT on the reconstructed source signal, so as to identify the fault according to the frequency in the amplitude spectrum of the separated signal.

## 5. The Simulation Analysis

The availability of the proposed algorithm (SMF−DPC−OMP) is verified by simulation signals. Simulation signals simulate the compound faults signal of bearing inner and outer rings. Random noise signals with positive and negative amplitudes of 1 and 3 are added to the experiment, respectively. The source signal 1 is a shock pulse generated by the Formula (15) cyclically, and every 128 points cycles to generate a shock pulse, and the source signal 2 is a simple sinusoidal signal generated by the Formula (16). The shock pulse frequency of source signal 1 is 100 Hz, and the sinusoidal signal frequency of source signal 2 is 45 Hz, and the amplitude is 1. The SNR of source signal 1 is −4 dB, and that of source signal 2 is −3.45 dB.

(14) $$s_{1}(t)=e^{-\alpha t}\sin(2\pi ft)$$

(15) $$s_{2}(t)=\sin(2\pi ft)$$

**Figure 4:**
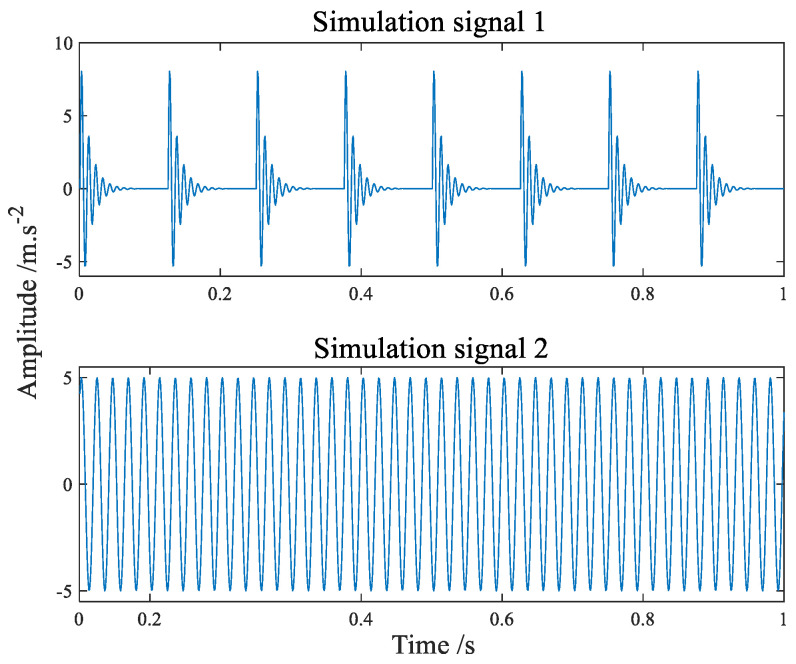
The time−domain waveform of simulation signals.

**Figure 5:**
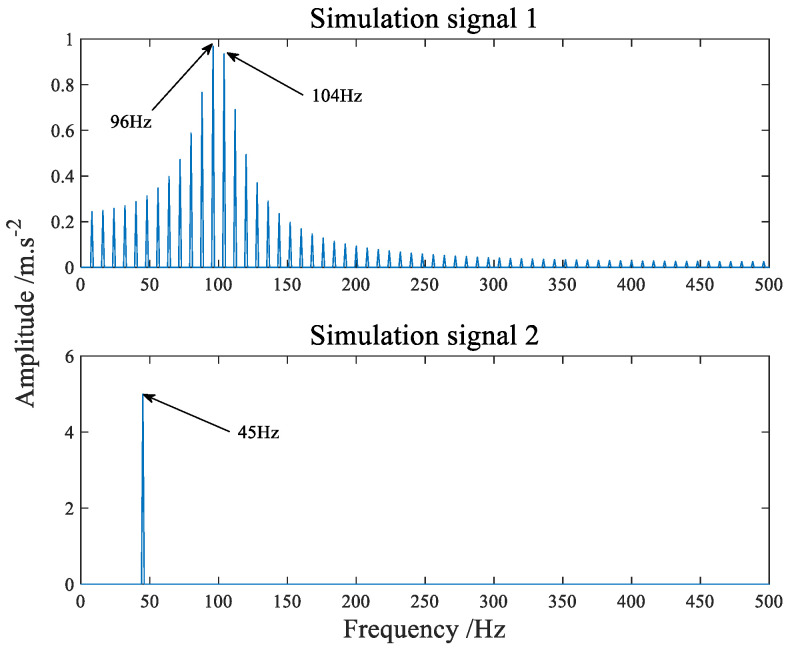
The amplitude spectrum of simulation signals.

**Figure 6:**
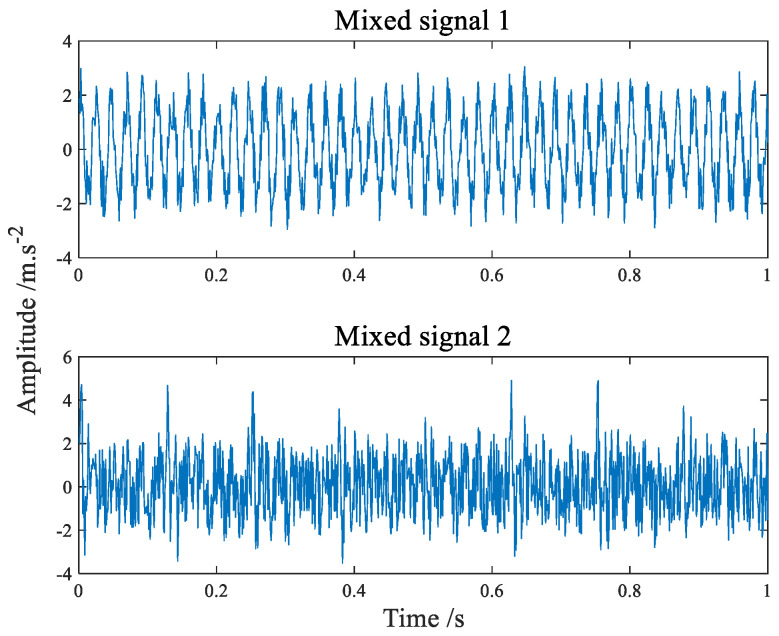
The time−domain waveform of mixed signals.

**Figure 7:**
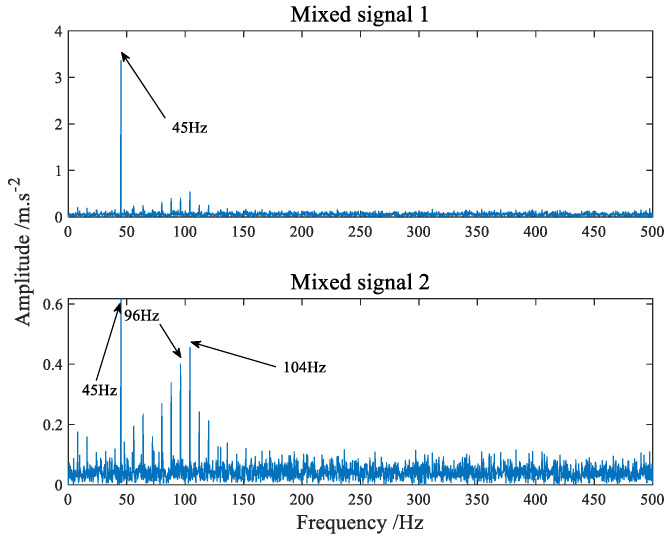
The amplitude spectrum of mixed signals.

The time-domain waveform of the simulation signals simulating the fault of the inner and outer ring of the bearing are shown in Figure 4 and Figure 5 shows its amplitude spectrum. Sampling frequency fs = 1042 Hz, sampling points *n* = 4096. A hybrid matrix is 2 × 3 matrix randomly generated by the computer. Figure 6 is the time waveform after adding noise and mixing by mixing matrix. Figure 7 is the result of the signal in Figure 6 passing through FFT. Through Figure 6 we can intuitively discover that the two signals have been mixed together. Both components in Figure 7 have frequencies of sinusoidal signals and impact signals, and the two signals are completely mixed with each other. If you notice the difference in amplitude, you will find that the first signal actually contains components of both signals in Figure 7. 

**Figure 8:**
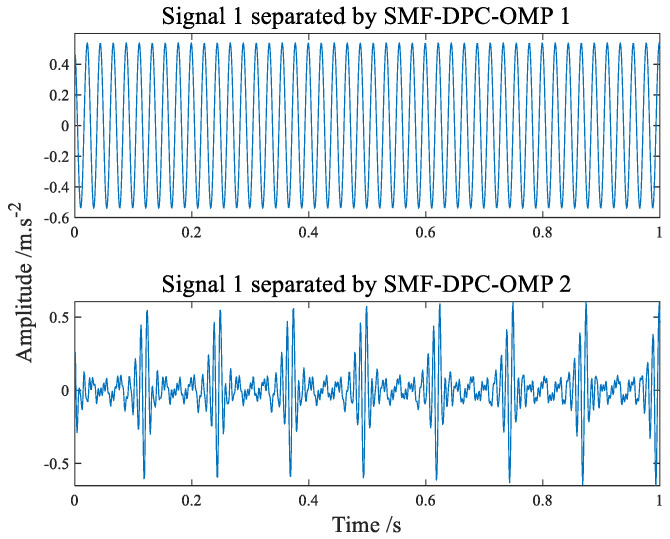
The time−domain waveform of separated signal.

**Figure 9:**
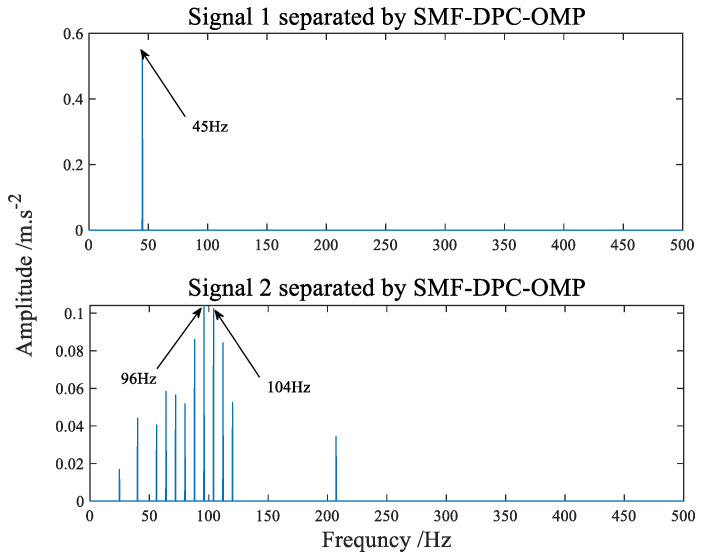
The amplitude spectrum of separated signal.

The separated signals recovered by the SMF−DPC−OMP algorithm are shown in Figure 8 and Figure 9, showing its time-domain waveform and amplitude spectrum, respectively. Whether from the time waveform or the amplitude spectrum, it can be immediately found that the source signal is very well separated. The first component diagram tersely shows the 45 Hz spectral line, which conforms to the frequency characteristics of source signal 2. The second component in Figure 9 has a side spectral line with an interval of 8 Hz near 96 Hz and 104 Hz, which conforms to the characteristic frequency of source signal 1. It can be seen from the analysis results that the algorithm can separate better and restore source signals well. As is known to all, blind separation has the problem of amplitude and order uncertainty, which makes the amplitude and order of source simulation signals and separated signals different but does not affect the characteristic frequency analysis and the effectiveness of the algorithm.

## 6. Experiment Verification

### 6.1. The Comparison Experiment of Different Algorithms under the Condition of Constant SNR

There is a lot of background noise in the down-to-earth environment, so the availability of the proposed algorithm is verified by the measured composite fault signals of rolling bearing. The vibration and fault simulation test platform of QPZZ−II rotary machinery is used to simulate the fault of rolling bearings. Relevant parameters of NU205 fault rolling bearing are shown in Table 1.

According to the bearing parameters of Table 1, we calculate each fault defect frequency. When the speed of the motor is 800 r/min, that is, the frequency of $f_{r}$ is 13.33 Hz, The characteristic frequency of bearing inner ring fault is 95.38 Hz, the outer ring fault defect frequency is 64.61 Hz.

**Figure 10:**
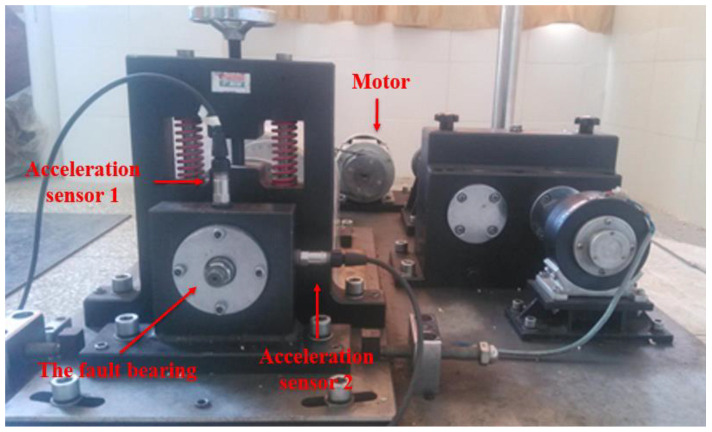
The test bench and microphone arrangement.

**Figure 11:**
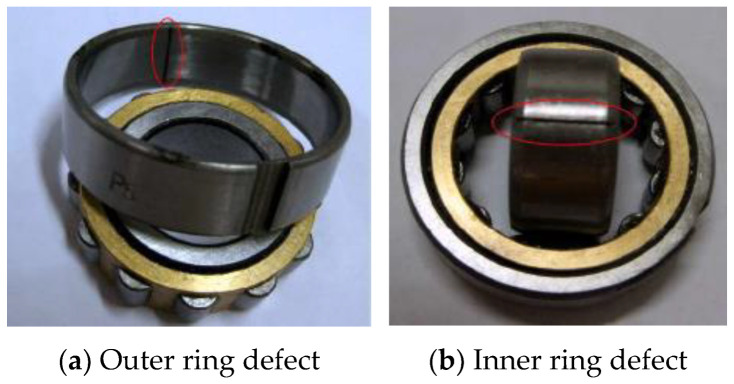
Physical type of rolling gear failure.

The acquisition system consists of the NI Signal Express acquisition module and the NI-9234 four-channel acquisition card. The sampling frequency is set to 8192 Hz and the sampling points is 8192. Frequency interval Δf=fs/N=1 Hz. Two pairs of acceleration sensors are vertically installed on the bearing seat in horizontal and vertical directions to obtain bearing vibration signals. The physical layout of the test bench and acceleration sensor is shown in Figure 10. The experimental analysis data come from sensors 1 and 2 identified in Figure 10. The fault type in this experiment is a compound fault of inner and outer ring. At the same time, to acquire a better sense of the shape, location and size of the bearing compound fault, the physical diagram of the faulty bearing is shown in Figure 11. The bearing fault is processed by wire cutting, and the size of the fault is 15 mm × 0.5 mm × 0.5 mm. In terms of parameter setting, due to the large difference in the number of horizontal and vertical coordinates of the vibration signal, after some tests, it is more appropriate to take the truncation error of 0.375. The OMP algorithm can complete the reconstruction task when the number of cycles is 40 times.

**Figure 12:**
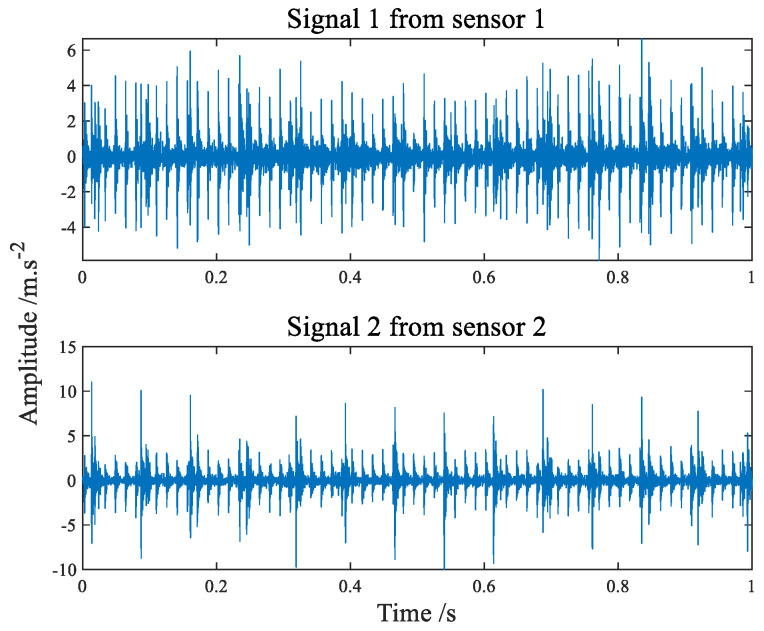
The time−domain waveform of composite fault signals.

**Figure 13:**
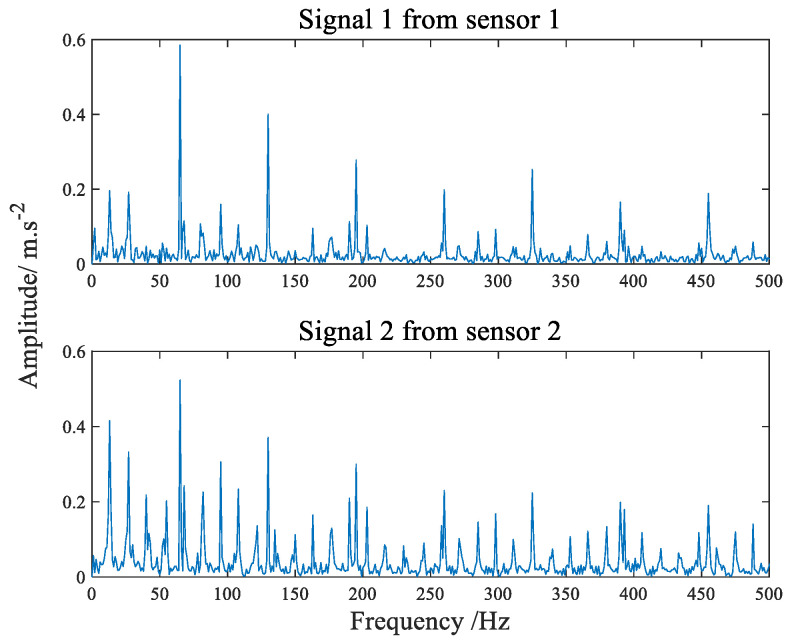
The envelope spectrum of composite fault signals.

**Figure 14:**
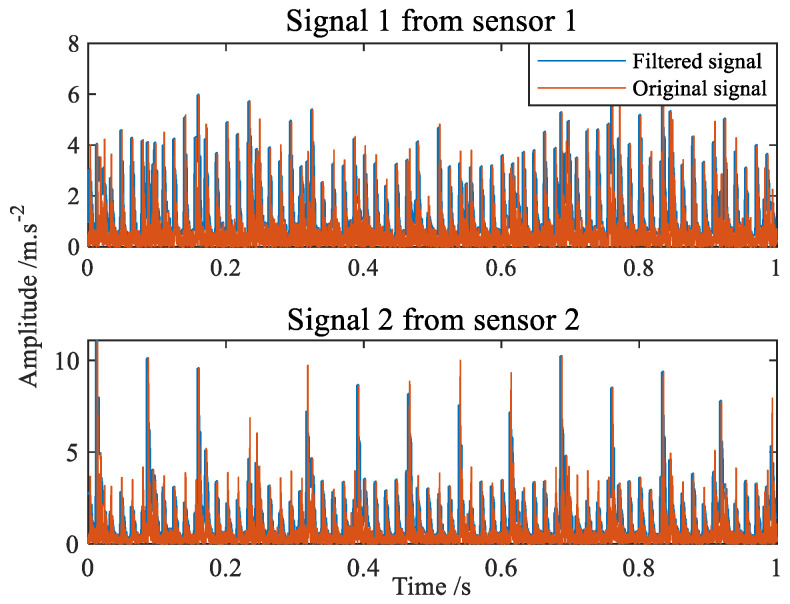
Signal contrast diagram before and after filtering.

Figure 12 shows the time-domain waveform diagram of compound faults of bearing inner and outer rings. The envelope spectrum can be seen in Figure 13. It is apparent from the envelope spectrum that the characteristic frequency components of the inner and outer ring composite faults are completely mixed together, making it difficult to judge the faults. The comparison before and after filtering with the improved filter is shown in Figure 14. The advantage of the improved filter over the straight line has been described in Section 4.1 and will not be repeated here. 

**Figure 15:**
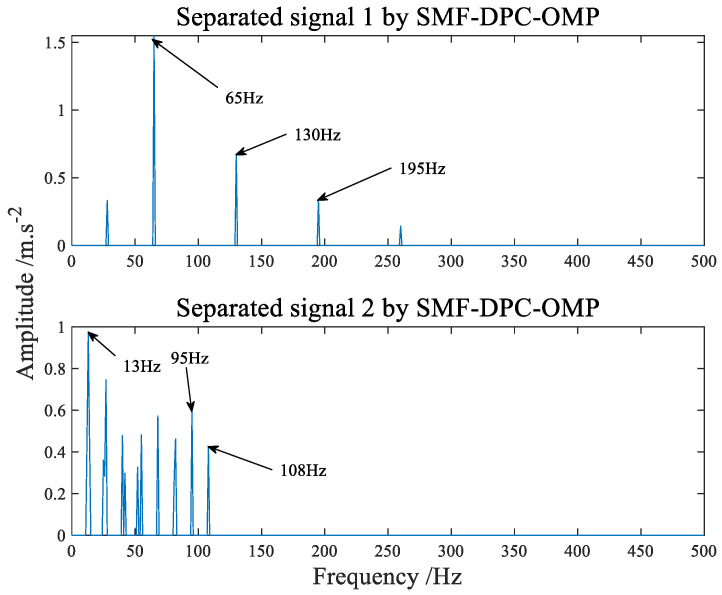
The frequency domain spectrum of separated signal processed by SMF−DPC−OMP.

**Figure 16:**
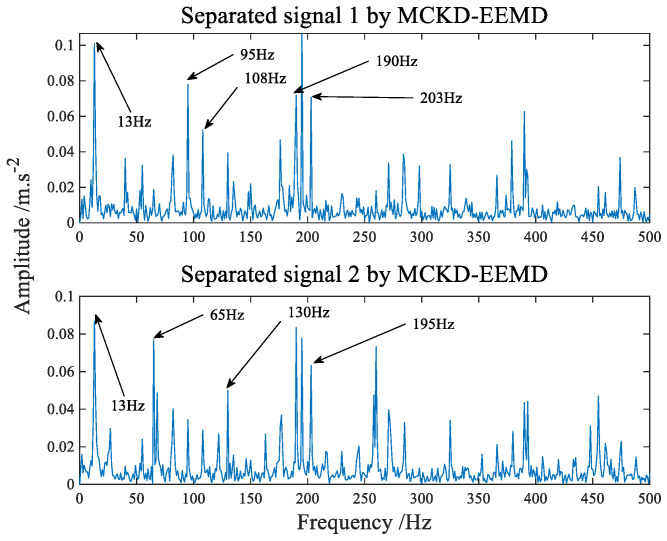
The frequency domain spectrum of separated signal processed by MCKD−EEMD.

The analysis results of SMF−DPC−OMP are shown in Figure 15, in which it can be seen that the composite faults have been successfully separated. Compared with Figure 16, the side frequency components are less, and the most pregnant thing is that there is no thin spectrum line in Figure 15. The first separation signal in Figure 15 clearly and cleanly displays 65 Hz, 130 Hz, 195 Hz and other spectral lines, and the results are consistent with the calculated outer ring fault defect frequency (64.61 Hz) or its frequency double. In addition, the second separated signal in Figure 15 contains key spectral lines of 13 Hz, 95 Hz, and 190 Hz. The bearing rotation frequency is 13 Hz. There are side frequency components with a rotation frequency of 13 Hz on both sides of 95 Hz and 190 Hz. On balance, the results are in line with the calculated inner ring failure frequency (95.38 Hz), in accordance with the inner ring fault characteristics.

**Figure 17:**
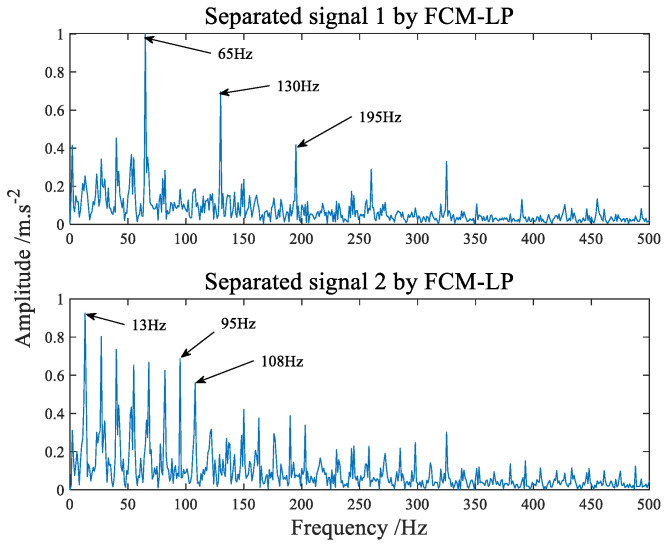
The frequency domain spectrum of separated signal processed by FCM−LP.

In order to verify the effectiveness of the proposed method, two methods are chosen here as comparison to reflect the advantages of the proposed algorithm. Firstly, a novel blind separation algorithm based on the combination of MCKD and EEMD [40] is used for comparison. The spectrum of composite fault signals separated by MCKD−EEMD algorithm is shown in Figure 16. From the observation of Figure 16, it is not difficult to find that blind separation of composite faults cannot be effectively realized by this algorithm. Then the algorithm of Fuzzy C−means clustering and linear programming (FCM−LP) [39] is selected, which is the common algorithm of traditional blind source separation. The spectrum of composite fault signals separated by FCM−LP algorithm is shown in Figure 17, although it can basically realize the separation of bearing inner and outer ring faults, but there are still a lot of side frequencies and thin lines.

### 6.2. The Validation Experiment of the Algorithm in this Paper under Different SNR Conditions

Taking into account the actual production, the noise in the environment cannot be as small as the noise in the laboratory, and the noise component of the experimentally collected signal in Section 6.1 is low. By adding different levels of noise to the collected signal, the signal containing different levels of noise can be obtained. These signals are used to test the feasibility of the proposed algorithm in this paper. In this section, the noise added to the signal ranges from 6 dB to −6 dB. Since the operation of adding noise from 6 dB to 0 dB has little influence on data, it will not be discussed in this chapter. The range of noise attached to the signal is from 0 dB to −6 dB is as described below.

**Figure 18:**
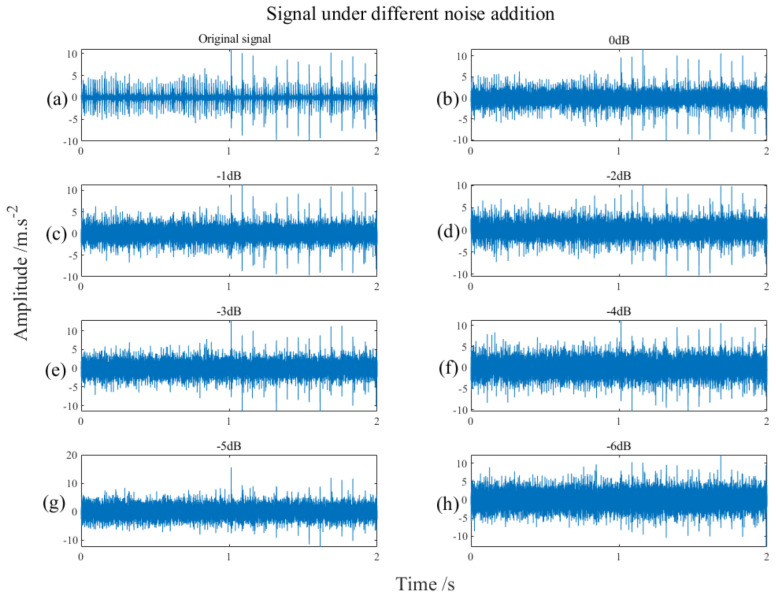
Experimental signals with different levels of noise, (**a**) is the case without adding noise, and (**b**–**h**) is the case with different levels of noise from 0 dB to −6 dB respectively.

The signals in Figure 18 are two sets of signals spliced to form a set of signals, so that readers can see the noise of signals more completely. Figure 18a shows the signal collected directly from the simulated failures test stand without additional noise, and Figure 18b–h are additional noises of different degrees. It is not difficult to see from Figure 18 that as the noise component increases, the impact component of the signal gradually becomes inconspicuous. The impact is not obvious, which is more in line with the fact that the noise in the actual production environment has a relatively strong impact.

**Figure 19:**
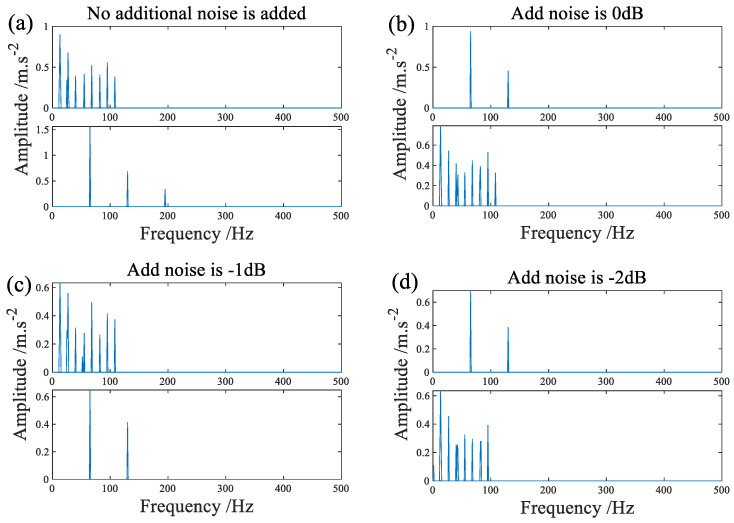
The signal is processed by SMF−DPC−OMP after additional noise, (**a**) is the case without adding noise, (**b**–**d**) is the case where 0 dB, −1 dB, and −2 dB of noise are added, respectively.

**Figure 20:**
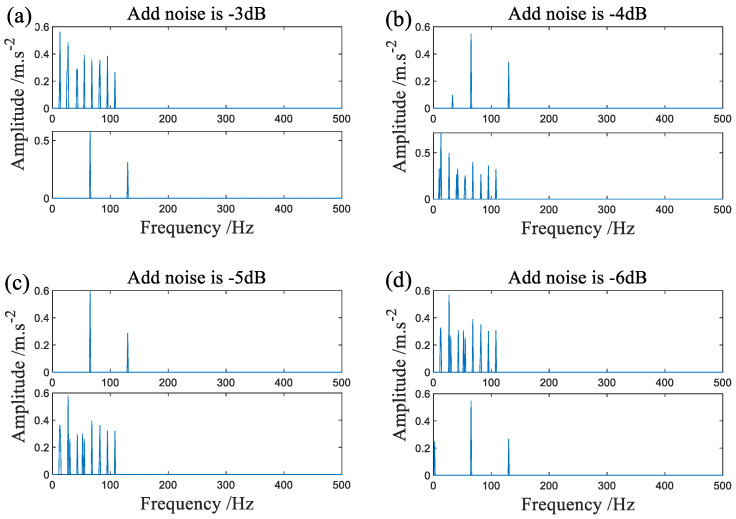
The signal is processed by SMF−DPC−OMP after additional noise, (**a**–**d**) is the case where −3 dB, −4 dB, −5 and −6 dB of noise are added, respectively.

Figure 19a shows the result of direct signal acquisition on the test bed processed by the algorithm in this paper. Figure 19b–d and Figure 20a–d respectively show the processing results separated by SMF−DPC−OMP when different levels of noise are added.

After comprehensive analysis of Figure 18, Figure 19 and Figure 20, the proposed algorithm can achieve fault separation even with different levels of noise. At the same time, first-class spectral line definition and low time cost are maintained in the frequency domain. Through experiments, it is not difficult to find that the only parameter of the algorithm in this paper, namely the truncation error, does not change in the blind source separation of faults for signals with different noise levels, which reflects the anti-noise ability of the algorithm.

## 7. Algorithm Analysis

**Figure 21:**
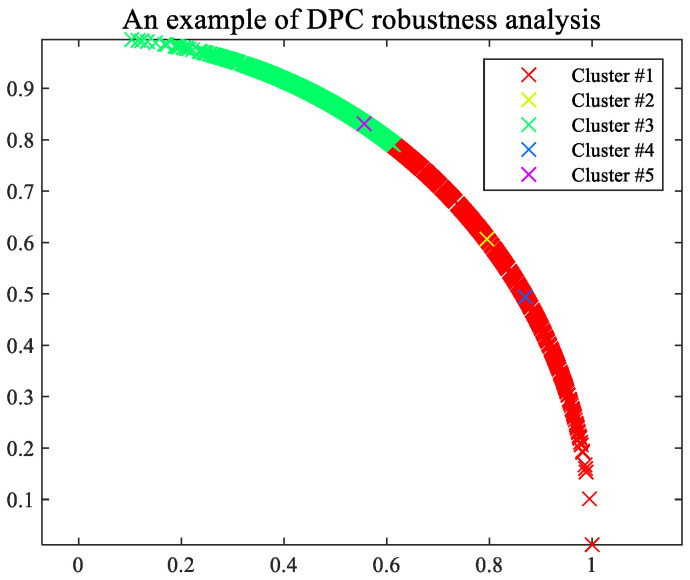
Schematic diagram of clustering effect.

The biggest innovation point of this paper is that BSS can be performed without prior knowledge of the number of fault sources, which is more consistent with the actual situation and has guiding significance for engineering applications. In addition, the algorithm has certain robustness to truncation error selection for the only parameter in clustering and can triumphantly complete blind source separation under the condition of improper parameter selection. For instance, improper selection leads to the clustering of vibration signals of two faults as multiple faults, as shown in Figure 21. Nevertheless, it can be seen from the clustering result graph that the inner points of other classes are far smaller than those of class 1 and 3, which can be excluded. Therefore, only cluster centers 1 and 3 can be selected. It is not difficult to find from Section 6.2 that when the noise of the signal changes within a certain range, it will not interfere with the selection of parameters.

Repeating 50 experiments for two fault signals and taking the average value, the time cost of FCM-LP is about 90.2 s, while that of SMF−DPC−OMP is 22.05 s. In comparison, the time cost is reduced by about 75%.

The shortcomings of the algorithm are mainly reflected in:

(1) Failure to apply to fault diagnosis under the condition of under-determination;
(2) The selection of cycle times of OMP algorithm is not self-adaptive;
(3) The selection of truncation error of DPC is obtained from many adjustments.

## 8. Conclusions

In complex mechanical structure and industrial environments, fault signals are often covered by various noises and the number of fault sources is unknown. In this paper, the improved morphological filtering algorithm is firstly proposed based on sinC function, and compared with a linear filter, the results show that the improved filtering algorithm can distinctly improve the signal-to-noise ratio. Then, utilizing the strong point of density peak clustering algorithm, the complex fault diagnosis is realized when the number of sources is unknown, and this advantage has some practical implications. Finally, compressed sensing and reconstruction are completed in the frequency domain to suppress fine side frequency and interference components while retaining the characteristic frequency containing fault information. The results are concise and clear. The feasibility of the proposed algorithm is verified by the separation of the acceleration vibration signals of the two-channel rolling bearing from the experimental simulation and the actual acquisition. Experimental results show that the time cost of the proposed method is about 75% higher than the FCM-LP algorithm. The future work of this paper will focus on fault extraction under underdetermined conditions, or further extend the algorithm to be applied to fault diagnosis of rotating machinery acoustic signals.

## Figures and Tables

**Table 1:** Parameters of example toroidal drive system.

Name	Values	Units
Inner diameter-*d*	25	mm
Outside diameter-*D*	52	mm
Inner ring width-*B*	15	mm
Pitch circle diameter-*d*′	39	mm
Diameter of rolling body-*D*_1_	7.5	mm
Number of rolling bodies-*Z*	12	/
The contact angle-*α*	0	*x*°
Motor speed-*n*	800	r/min

## Data Availability

Not applicable.
